# The balance of proangiogenic and antiangiogenic VEGFA isoforms regulate follicle development

**DOI:** 10.1007/s00441-012-1330-y

**Published:** 2012-02-10

**Authors:** Renee M. McFee, Timothy G. Rozell, Andrea S. Cupp

**Affiliations:** 1 Department of Animal Science, Kansas State University, Manhattan, KS 66506 USA; 2 Department of Animal Science, University of Nebraska-Lincoln, A224i Animal Science Building, 3800 Fair Street, Lincoln, NE 68583-0908 USA

**Keywords:** VEGFA, Isoforms, Ovary, Follicle, Vasculature

## Abstract

Vascular endothelial growth factor A (VEGFA) has been extensively studied because of its role in follicular development and is a principal angiogenic factor essential for angiogenesis. Since vascularization of the theca layer increases as follicles progress in size through preantral and antral stages, VEGFA might influence follicle growth via the regulation of angiogenesis. However, VEGFA might also influence follicular development through nonangiogenic mechanisms, since its expression has been localized in nonvascular follicles and cells. Alternative mRNA splicing of eight exons from the *VEGFA* gene results in the formation of various VEGFA isoforms. Each isoform has unique properties and is identified by the number of amino acids within the mature protein. Proangiogenic isoforms (VEGFA_XXX) are encoded by exon 8a, whereas a sister set of isoforms (VEGFA_XXXB) with antiangiogenic properties is encoded by exon 8b. The antiangiogenic VEGFA_XXXB isoforms comprise the majority of VEGFA expressed in most tissues, whereas expression of the proangiogenic VEGFA isoforms is upregulated in tissues undergoing active angiogenesis. Although proangiogenic and antiangiogenic isoforms can now be distinguished from one another, many studies evaluating VEGFA in ovarian and follicular development up to now have not differentiated proangiogenic VEGFA from antiangiogenic VEGFA. Experiments from our laboratory indicate that proangiogenic VEGFA promotes follicle recruitment and early follicular development and antiangiogenic VEGFA inhibits these processes. The balance of proangiogenic versus antiangiognic VEGFA isoforms is thus of importance during follicle development. Further studies are warranted to elucidate the way that this balance regulates follicular formation and progression.

## Introduction

Angiogenesis is the term used to describe the formation of new vessels from the remodeling and expansion of the existing vascular network. This process involves both proliferation and migration of endothelial cells and can lead to the vascularization of previously avascular tissues (Patan 2000; Shimizu et al. 2003b). Ovarian and follicular vasculature enables the delivery of nutrients, oxygen and systemic hormones and the release of ovarian hormones (Robinson et al. 2009; Shimizu et al. 2003b). Because of recurring cyclical changes and the development of follicles, continued angiogenesis is essential for these ovarian functions.

Follicle assembly and initial recruitment of primordial follicles begins near the corticomedullary border and progresses outward to the periphery (Rajah et al. 1992; van Wezel and Rodgers 1996). At this stage of development, the major ovarian vessels are only located within the medulla (Brennan et al. 2002). Primordial, primary and early secondary follicles are not directly supplied with vasculature but are able to receive nutrients and oxygen by passive diffusion from vessels in the surrounding stroma (Robinson et al. 2009; Shimizu et al. 2003b; Suzuki et al. 1998). Therefore, follicle assembly and growth at these stages might be influenced by close association with the vasculature and the associated delivery of mediating factors.

In order for follicles to progress past these early developmental stages, an individual capillary network needs to form around each follicle (Suzuki et al. 1998). Vascularization is first visible in follicles that contain four layers of granulosa cells (Wulff et al. 2001). All capillaries are located outside of the basement membrane of the follicle and granulosa cells remain avascular throughout follicle development (Suzuki et al. 1998; Tamanini and De Ambrogi 2004). Proliferation of the theca layer significantly increases from the early to late secondary follicle stage and approximately one quarter of these proliferating cells are endothelial cells (Wulff et al. 2001). A significant increase in vasculature has been demonstrated during preantral follicle development in pigs (Martelli et al. 2006) and follicular blood flow has been shown to be necessary for the continued growth of small antral follicles in cows (Acosta et al. 2005). In rabbits, both vasodilation and extension of thecal capillaries support the increase of blood flow during follicle growth (Macchiarelli et al. 1993).

Vascular changes continue throughout preovulatory development. In women, blood flow to the apical aspect of preovulatory follicles has been demonstrated to decrease, whereas flow to the basal and lateral follicle walls remains unchanged. Presumably, this change in blood flow is necessary for eventual follicle rupture (Brännström et al. 1998). Furthermore, subcutaneous injection of adult rats with TNP-470, an angiogenic inhibitor previously used to reduce tumor growth, not only causes a reduction in follicular angiogenesis but also prevents ovulation (Iijima et al. 2005).

In addition to regulating follicle development, alterations in the follicular vasculature might also be involved in follicular degeneration. Early indicators of follicular atresia include a reduction in follicle vascularity and decreased DNA synthesis in endothelial cells within the theca layer (Greenwald 1989). In sheep, the capillary network in the theca layer has been shown to undergo a significant reduction as atresia progresses (Hay et al. 1976). In humans, the capillaries within atretic follicles are thin, have reduced branching and are not uniformly distributed (Macchiarelli et al. 1993).

Angiogenesis is a highly regulated process that involves control from both proangiogenic and antiangiogenic factors. Principal proangiogenic factors include vascular endothelial growth factor A (VEGFA), fibroblast growth factor 2, members of the platelet-derived growth factor family and angiopoietins (Carmeliet 2000; Robinson et al. 2009). Of these factors, VEGFA has been extensively studied in regard to its role in angiogenic regulation (Ferrara 2004; Ferrara and Davis-Smyth 1997) and has also been investigated as a factor involved in follicular development. For example, intraperitoneal injection of mature rats with VEGFA results in increased numbers of preovulatory follicles, decreased numbers of atretic follicles and increased numbers of ovulated oocytes compared with control rats (Iijima et al. 2005). In contrast, the subcutaneous administration of truncated versions of the VEGFA receptors, KDR (kinase insert domain protein receptor) and FLT1 (FMS-like tyrosine kinase 1), fused to IgG (Trap compounds that inhibit VEGFA) to adult marmoset monkeys during the follicular phase inhibits ovulation and results in twice as many atretic follicles compared with control monkeys (Wulff et al. 2002). VEGFA might also be necessary for increased oocyte competence, since the concentration of VEGFA protein in follicular fluid from antral follicles is significantly greater in women that become pregnant after in vitro fertilization compared with those that do not (Zhao et al. 2010).

## VEGFA and its receptors

VEGFA (also known as VEGF) has been shown to promote migration, proliferation and tube formation in endothelial cells (Patan 2000) and is a member of the platelet-derived growth factor and vascular endothelial growth factor family. This family also includes placenta growth factor, VEGFB, VEGFC and VEGFD (Ferrara 2004). VEGFA is essential for both vasculogenesis and angiogenesis. Loss of VEGFA in mouse models leads to severe vascular abnormalities and is embryonic lethal between 11-12 days postcoitus (dpc; Carmeliet et al. 1996; Ferrara et al. 1996). Messenger RNA expression for VEGFA is prominently stimulated by hypoxia but VEGFA expression is also upregulated by several other factors, such as platelet-derived growth factor, insulin-like growth factor-1, tumor necrosis factor-alpha, fibroblast growth factor, transforming growth factor alpha and beta and epidermal growth factor (Ferrara 2004; Robinson and Stringer 2001).

Two tyrosine kinase receptors bind VEGFA with high affinity: FLT1 (also referred to as VEGFR1) and KDR (also referred to as VEGFR2). Both of these receptors have seven extracellular immunoglobulin-like domains, a single transmembrane region and an intracellular tyrosine kinase sequence with a kinase insert domain (Ferrara 2004; Robinson and Stringer 2001). FLT1 was the first VEGFA receptor identified and has a high affinity for VEGFA; however, VEGFA binding results in only weak tyrosine phosphorylation and does not appear to induce a proliferative response (de Vries et al. 1992; Park et al. 1994; Seetharam et al. 1995; Waltenberger et al. 1994). Thus, FLT1 has been proposed to regulate VEGFA activity negatively by sequestering it and limiting its availability to bind KDR (Park et al. 1994). Mutant mice that lack FLT1 die between 8.5-9.5 dpc and display severe vascular disorganization and an increased number of endothelial progenitor cells (Fong et al. 1995, 1999). However, mutant mice that possess an intact VEGFA-binding region on FLT1 but lack a tyrosine kinase domain are able to develop fully and display normal vascular development (Hiratsuka et al. 1998).

Although FLT1 has a higher affinity for VEGFA than KDR, VEGFA binding to KDR induces stronger tyrosine phosphorylation (Waltenberger et al. 1994). KDR is believed to mediate most, if not all, of VEGFA’s regulation of endothelial cell proliferation and migration. Mutant mice that lack KDR die between 8.5-9.5 dpc and fail to develop organized blood vessels (Shalaby et al. 1995). Mutated VEGFA proteins that lack affinity for the KDR receptor fail to stimulate proliferation and migration of bovine endothelial cell cultures. In contrast, treatment of these cell cultures with VEGFA protein mutants that lack affinity for the FLT1 receptor stimulates endothelial cell proliferation similar to that induced by treatment with wild-type VEGFA protein (Keyt et al. 1996).

In addition to FLT1 and KDR, VEGFA has also been shown to bind to neuropilins. Mutant mice that lack neuropilin-1 (NRP1) die between 10.5-12.5 dpc with multiple vascular defects and mutant mice that overexpress NRP1 die at 17.5 dpc with excessive and dilated vasculature (Kawasaki et al. 1999; Kitsukawa et al. 1995). NRP1 appears to function as a coreceptor by presenting VEGFA to KDR. NRP1 has an extremely short intracellular domain and is unable to stimulate cellular responses in the absence of KDR (Soker et al. 1998). NRP1 has also been shown to bind directly to FLT1. Therefore, one of the mechanisms by which FLT1 negatively regulates VEGFA activity might be through competition for NRP1 binding (Fuh et al. 2000).

## VEGFA isoforms

**Fig. 1:**
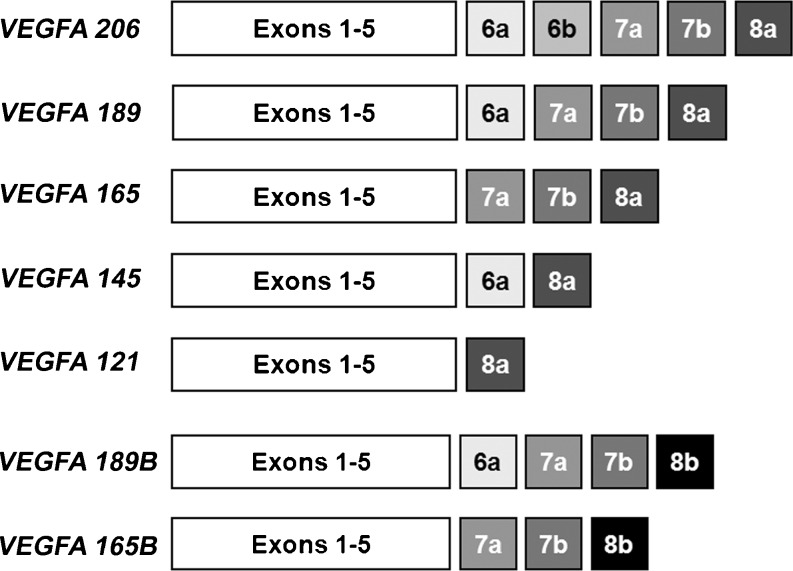
Alternate splicing of the human *vascular endothelial growth factor A* (*VEGFA*) gene results in different VEGFA isoforms. Each isoform is encoded by a specific set of exons and the resulting proteins are named by their unique number of amino acids. Exon 8a encodes the proangiogenic isoforms, whereas exon 8b encodes the antiangiogenic “B” isoforms

VEGFA is encoded by a single gene but various isoforms exist because of alternative mRNA splicing of eight exons (Fig. 1). The different isoforms are identified by their number of amino acids and each isoform has unique properties (Houck et al. 1991; Tischer et al. 1991). Of the predominant isoforms, VEGFA_121 (exons 1-5 and 8a; Fig. 1) is the shortest protein and is unable to bind heparin. VEGFA_165 (exons 1-5, 7 and 8a; Fig. 1) and VEGFA_189 (exons 1-5, 6a, 7 and 8a; Fig. 1) contain additional amino acid sequences encoded by exons 6 and 7. VEGFA_165 has moderate affinity for heparin because of the amino acid residues encoded by exon 7. VEGFA_189 has additional residues encoded by exon 6 and, thus, has a high affinity for heparin binding (Robinson and Stringer 2001). VEGFA_189 is almost entirely bound to either cell surfaces or the extracellular matrix (ECM), presumably via interactions with heparin-containing proteoglycans. Approximately 50%-70% of VEGFA_165 is bound to cells or the ECM, whereas VEGFA_121 is freely diffusible (Houck et al. 1992). NRP1 is able to bind to VEGFA_165 but not to VEGFA_121. NRP1 enhances the binding of VEGFA_165 to KDR and, thus, its regulation of the proliferation and migration of endothelial cells (Soker et al. 1998). Several other isoforms have been isolated in different cells and species, including VEGFA_115, VEGFA_145, VEGFA_162, VEGFA_183 and VEGFA_206 (Anthony et al. 1994; Cheung et al. 1995; Jingjing et al. 1999; Lange et al. 2003; Lei et al. 1998; Sugihara et al. 1998).

In addition to differences in amino acid length, VEGFA isoforms have been identified that originate from alternative splicing of exon 8. The previously described VEGFA isoforms are encoded by exon 8a (Fig. 1). Replacement of exon 8a with exon 8b generates a novel sister set of isoforms (VEGFA_XXXB) referred to as the “B” isoforms (Fig. 1). The VEGFA_XXXB isoforms only differ from the other VEGFA isoforms by the six amino acids located at the C’-terminal end and instead of being proangiogenic, the presence of residues encoded by exon 8b confers antiangiogenic properties (Bates et al. 2002; Cui et al. 2004; Harper and Bates 2008; Konopatskaya et al. 2006; Woolard et al. 2004).

In most normal adult tissues, the VEGFA_XXXB isoforms comprise at least half of the total VEGFA expressed. In normal tissues undergoing active angiogenesis, such as the placenta or in pathologic states, such as neoplasia, VEGFA_XXXB isoform expression is downregulated (Harper and Bates 2008; Woolard et al. 2004). For example, mRNA for the VEGFA_165B isoform has been detected in 94.4% of normal kidney samples but only in 22.2% of renal cell carcinoma samples from the same human patients (Bates et al. 2002). Similarly, VEGFA_XXXB isoforms comprise approximately 91% of the total amount of the VEGFA mRNA amplified from normal colorectal tissue but less than 55% of the total mRNA from colorectal tumor tissue from the same human patients (Varey et al. 2008). Differential expression of the proangiogenic versus antiangiogenic VEGFA isoforms is also evident in proliferative diabetic retinopathy. This condition develops from hypoxia-mediated blood vessel growth that extends from the retina into the vitreous chamber in human diabetic patients. The VEGFA_XXXB isoforms represent approximately 64% percent of the VEGFA protein isolated from the vitreous of non-diabetic human patients but only 12.5% of the VEGFA protein isolated from the vitreous in diabetic patients (Bevan et al. 2008). Therefore, alternate splicing of the VEGFA gene and the resulting ratio of increased proangiogenic versus antiangiogenic isoforms appears to be an important regulator of angiogenesis.

The VEGFA_XXXB isoforms were considered as being antiangiogenic shortly after their identification, because treatment with VEGFA_165B inhibited VEGFA_165-mediated proliferation and migration of cultured endothelial cells (Bates et al. 2002). One study demonstrated that, whereas VEGFA_165 could stimulate angiogenesis in rabbit corneas, VEGFA_165B did not stimulate angiogenesis and could even inhibit VEGFA_165-mediated corneal angiogenesis (Woolard et al. 2004). In another study, intraocular injections of VEGFA_165B resulted in a nearly 50% reduction in the area of hypoxia-induced retinal neovascularization in mice (Konopatskaya et al. 2006). In addition, melanoma cells expressing VEGFA_165B injected into nude mice produced significantly smaller tumors than melanoma cells expressing VEGFA_165 (Woolard et al. 2004).

The antiangiogenic properties of the VEGFA_XXXB isoforms are believed to be related to the inefficient stimulation of downstream signaling. VEGFA_165B has been shown to bind to KDR with similar affinity as VEGFA_165 but does not activate downstream signaling via KDR. Not only does treatment of human endothelial cells with VEGFA_165B result in less KDR phosphorylation than treatment with VEGFA_165 but VEGFA_165B-treated cells demonstrate similar phosphorylation as untreated cells. Treatment of these cells with both VEGFA_165 and VEGFA_165B also results in less phosphorylation than treatment with VEGFA_165 alone (Woolard et al. 2004). Another study has demonstrated that VEGFA_165B is able to induce phosphorylation by binding KDR but causes significantly less phosphorylation at certain KDR sites, including one of the positive mouse regulatory sites, Y1052. Furthermore, VEGFA_165B is not able to bind NRP1 and this might explain the ineffectiveness of these antiangiogenic VEGFA at signaling upon binding to KDR (Kawamura et al. 2008).

The various VEGFA isoforms are named for the number of amino acids that comprise each protein; however, the number of amino acids for similar isoforms can vary between species. For example, the predominant proangiogenic isoform in humans has 165 amino acids (VEGFA_165) but the corresponding isoform in mice, rats and cattle consists in only 164 amino acids (VEGFA_164; Bacic et al. 1995; Breier et al. 1992; Robinson and Stringer 2001; Shimizu and Miyamoto 2007). The antiangiogenic isoform that has been the most extensively studied is human VEGFA_165B, which has the same number of amino acids as its proangiogenic counterpart, VEGFA_165 (Bates et al. 2002; Harper and Bates 2008). We have sequenced the mRNA for *VEGFA_164B* (GenBank accession number EU017524.1) from bovine granulosa cells. Based on the predicted amino acid sequence, the antiangiogenic isoforms appear to have the same number of amino acids as the proangiogenic isoforms in the bovine. However, we have also sequenced the mRNA for *Vegfa_165b* from rat ovaries and, based on the predicted amino acid sequence, the antiangiogenic isoforms appear to have an additional amino acid compared with their respective proangiogenic isoforms in rats (Artac et al. 2009). To avoid confusion, all VEGFA isoforms will be referred to, in this paper, by using the number of amino acids found in humans (e.g., VEGFA_121, VEGFA_164, VEGFA_189, VEGFA_165B) regardless of the species being discussed.

## Establishment of the primordial follicle pool

In mammals, the oocyte population is primarily believed to be nonrenewable and the number of primordial follicles formed during fetal or early perinatal life is the factor that limits the reproductive life span (Hansen et al. 2008; McLaughlin and McIver 2009; Perez et al. 1999). A decrease in the number of primordial follicles can result in reduced fertility, premature ovarian failure, or sterility. Exposure of fetal rats to gamma-irradiation severely depletes the number of oocytes and premature ovarian failure occurs at approximately 6 months of age despite the normal onset of puberty and initial fertility (Mazaud et al. 2002). In addition, the treatment of fetal rats with busulphan results in a dose-dependent reduction in the number of primordial follicles that develop in treated animals compared with controls and severely depleted rats will exhaust their supply of follicles early in adulthood (approximately 60 days of age; Hirshfield 1994).

During follicle assembly, approximately one third of the oocytes are arrested at the diplotene stage of the first meiotic division and are incorporated into primordial follicles, whereas the remaining two thirds of germ cells are lost through apoptosis (Pepling and Spradling 2001). Overexpression of the anti-apoptotic factor, BCL2 (B-cell leukemia/lymphoma 2), in mouse ovaries leads to an increase in the initial primordial follicle pool but this difference is lost by 2 months of age (Flaws et al. 2001). Interestingly, loss of BCL2 function in mice results in a dramatic reduction in the number of normal primordial follicles and in the development of numerous primordial follicle-like structures that contain granulosa cells but lack an oocyte (Ratts et al. 1995). Furthermore, deletion of the pro-apoptotic factor, BAX (BCL2-associated X protein), in mice also leads to an increase in the initial primordial follicle pool and this surplus is maintained into late adult life, resulting in sustained ovarian function to nearly 2 years of age (Perez et al. 1999). Proangiogenic VEGFA isoforms are possibly involved in stimulating the increase in BCL2 that promotes germ cell survival. Although studies in females are lacking, experiments utilizing 4-week-old bovine testes have demonstrated a role for VEGFA in regulating germ cell death. Increased numbers of germ cells together with increased mRNA levels of *BCL2* relative to *BAX* are seen after VEGFA_165 treatment (Caires et al. 2009).

Previous studies in our laboratory have demonstrated immunohistochemical staining for VEGFA protein and VEGFA_XXXB isoforms in oocyte cysts and pregranulosa cells of primordial follicles in embryonic and postnatal rat ovaries. In addition, VEGFA_XXXB isoforms are also localized to the oocytes of primordial follicles (Artac et al. 2009; McFee et al. 2009). The VEGFA receptors, FLT1 and KDR, are localized to oocyte cysts and to both pregranulosa cells and oocytes of primordial follicles (McFee et al. 2009). Other studies have demonstrated weak VEGFA immunostaining of ooctyes in primordial follicles from human ovaries (Otani et al. 1999) and KDR expression has been localized to oocytes of primordial follicles from goat ovaries (Bruno et al. 2009).

**Fig. 2:**
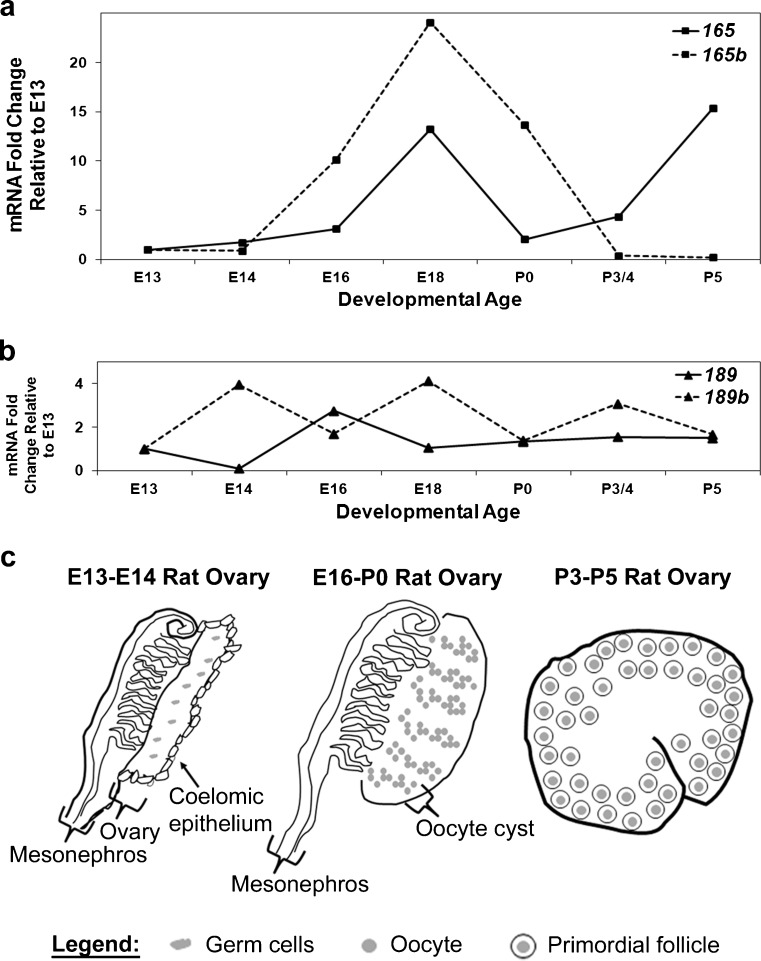
Quantitative reverse transcription with the polymerase chain reaction was conducted to detect mRNA levels for *Vegfa_165*, *Vegfa_165b*, *Vegfa_189* and *Vegfa_189b* in rat ovaries from embryonic day 13 (E13) through postnatal day 5 (P5) of ovarian development. *Gapdh* (*D-glyceraldehyde-3-phosphate dehydrogenase*) was used as an endogenous control to account for differences in starting material. These data are the result of at least three different pools of tissue from each age group. The mean normalized values obtained for E13 have been set at 1 and the values for the other developmental ages are presented as a fold-change from E13. Therefore, values greater than 1 indicate increased mRNA levels and values less than 1 indicate reduced mRNA levels in comparison with E13 (**a**, **b**). The primary morphologic stages that occur during development of the rat ovary from E13-P5 are presented in **c**

Our laboratory has also demonstrated mRNA expression for the *Vegfa_121*, *Vegfa_165*, *Vegfa_189*, *Vegfa_165b* and *Vegfa_189b* isoforms in rat ovaries during late embryonic and early postnatal development (Artac et al. 2009; McFee et al. 2009). In contrast to male gonadal development, distinct morphologic structures do not appear in the developing rat ovary until ovigerous cords form at the end of gestation at approximately embryonic day 16-18 (∼E16-E18). Oocytes are contained within these cord-like structures until postnatal day 1 (P1) when primordial follicles begin to assemble (Fröjdman et al. 1993; Hirshfield and DeSanti 1995; Rajah et al. 1992). Expression of mRNA for the predominant proangiogenic isoform, *Vegfa_165* and its comparable antiangiogenic isoform, *Vegfa_165b* (Artac et al. 2009; McFee et al. 2009) significantly increases between E13 and E18 in the rat ovary (Fig. 2a) and the timing of this upregulation coincides with ovigerous cord formation. Therefore, the upregulation of angiogenic *Vegfa* and the downregulation of antiangiogenic *Vegfa_xxxb* isoforms might help to promote follicle assembly.

Systemic (IP) administration of adult mice with an antibody designed to neutralize VEGFA reduces the number of primordial follicles by approximately 50% without having an effect on primary or secondary follicles. Similar results are seen with intrabursal administration of the same antibody to prepubertal (6- to 8-weeks-old) mice. Intrabursal administration of a KDR antibody also reduces primordial follicle numbers in prepubertal mice but injection with a FLT1 antibody has no effect. Although these differences in primordial follicles numbers are lost between 30 days (immature mice) or 6 months (adults) after treatment (Roberts et al. 2007), these data suggest that VEGFA and KDR play a role in the maintenance of the primordial follicle pool.

## Recruitment of primordial follicles

The duration of a female’s reproductive lifespan is determined not only by the number of primordial follicles that are formed but also by the rate at which this pool of quiescent follicles is depleted. Primordial follicles can remain quiescent or arrested in development for months to years to decades, depending on the female’s normal reproductive lifespan. A primordial follicle is defined as an individual oocyte surrounded by a single layer of pregranulosa cells. A primary follicle is characterized by a single layer of granulosa cells (Kezele et al. 2002; Parrott and Skinner 1999; Smitz and Cortvrindt 2002). The transformation of flattened pregranulosa to cuboidal granulosa cells is a relatively slow process (taking longer than 4 months in humans) and follicles are not considered to be actively growing until they have reached the primary stage (Gougeon and Chainy 1987; Smitz and Cortvrindt 2002). This transition is also an irreversible process and a follicle will continue to grow until its eventual demise, either through atresia or ovulation.

Initial follicle recruitment begins near the corticomedullary border (Hirshfield 1992; van Wezel and Rodgers 1996). This is similar to the pattern that occurs during follicle formation. Close association of recruited follicles to the medulla might be related to these follicles being exposed to factors diffusing from the medullary vasculature. The total number of follicles within the pool has also been proposed to influence the rate at which primordial follicles are recruited. Administration of busulphan to pregnant rats to destroy primordial germ cells in developing fetuses has revealed an inverse correlation between the number of primordial follicles in the initial pool and the rate at which these follicles are recruited to the growing pool (Hirshfield 1994). Morphometric studies have also demonstrated an accelerated rate of follicle recruitment in women as they approach menopause and their supply of primordial follicles dwindles (Faddy et al. 1992; Gougeon et al. 1994; Hansen et al. 2008; Richardson et al. 1987).

Several studies have investigated possible factors that are involved in the regulation of primordial follicle recruitment. Ovarian microarray analysis has revealed the upregulation of 148 genes and the downregulation of 50 genes in PO rat ovaries cultured for 1 week which contain predominately primary follicles when compared with freshly isolated P4 ovaries containing predominately primordial follicles. A high proportion of primary follicles are found in cultured ovaries, because the primordial to primary transition occurs at a faster rate in culture (Kezele and Skinner 2003). One of the 148 genes upregulated in this study was VEGFA. Quantitative polymerase chain reaction analysis has also identified the same increase in *Vegfa* gene expression (Kezele et al. 2005).

One might assume that this upregulation of VEGFA has a role in promoting the recruitment of primordial follicles into the growing follicle pool. However, a similar study has produced conflicting results. The culture of P4 rat ovaries with AMH reduces the number of primordial follicles that transition to the primary stage; however, microarray analysis of these cultured ovaries has revealed an upregulation of VEGFA (Nilsson et al. 2007). The results from this study suggest that the upregulation of VEGFA has a role in suppressing the recruitment of primordial follicles. One reason for the conflicting results is that these studies do not distinguish which VEGFA isoforms are upregulated. Proangiogenic VEGFA isoforms might be upregulated during the primordial to primary follicle transition and the antiangiogenic isoforms are possibly upregulated when this process is suppressed. Indeed, studies in our laboratory with developing rat ovaries have shown that mRNA expression for both *Vegfa_165* and *Vegfa_165b* increases dramatically from E13 to E18. After birth, mRNA expression of both of these isoforms declines (Artac et al. 2009; McFee et al. 2009). However, *Vegfa_165b* expression dramatically declines to levels less than those measured at E13, whereas *Vegfa_165* expression rebounds from P0 to P5 (Fig. 2a). No trends are apparent for the mRNA expression for either *Vegfa_189* or *Vegfa_189b* during these developmental time points (Fig. 2b). Therefore, these data suggest that the upregulation of angiogenic *Vegfa* and the downregulation of antiangiogenic *Vegfa_xxxb* isoforms might help to promote follicle assembly and early follicular recruitment.

Expression of VEGFA isoforms might be related to angiogenesis of the ovary and/or follicles; however, both primordial and primary follicles are avascular. Despite being avascular, we have located VEGFA, VEGFA_XXXB isoforms, FLT1 and KDR in pregranulosa/granulosa cells of both of these follicle types by using immunohistochemical staining of postnatal rat ovaries. Protein expression for FLT1 and KDR has also been localized to oocytes within these follicles (Artac et al. 2009; McFee et al. 2009). Other studies have demonstrated weak VEGFA immunostaining of oocytes in primary follicles from adult rat ovaries (Celik-Ozenci et al. 2003). In goat ovaries, KDR expression has been localized to oocytes of primordial and primary follicles and to granulosa cells of primary follicles (Bruno et al. 2009). These data provide further support that VEGFA signaling plays a role in the maintenance and/or activation of primordial follicles.

**Fig. 3:**
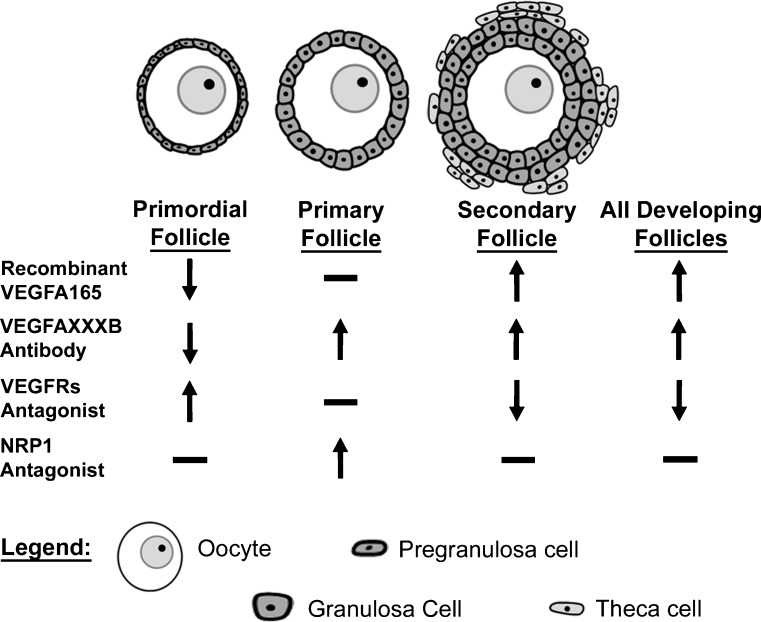
One ovary from postnatal day 3/4 rat was cultured for 2 weeks with either recombinant VEGFA_165, antibodies to VEGFA_XXXB isoforms, an inhibitor of KDR and FLT1 (VEGFA receptors), or an inhibitor to NRP1. The paired ovary from each rat was cultured without treatment to serve as a control. The mean number of follicles at each stage of development was calculated as a percent of total follicles, and these percentages were compared between treated and control ovaries (*↑* significant increas in the percent of follicles at each stage in treated ovaries in comparison with controls, *↓* significant decrease in the percent of follicles at each stage in treated ovaries in comparison with controls, *-* no difference in the percent of follicles at each stage in treated ovaries in comparison with controls). All developing follicles include early primary, primary, transitional, and secondary follicle stages

Experiments in our laboratory utilizing P3/4 rat ovary cultures also support a role for VEGFA isoforms in initial follicle recruitment. Treatment with a VEGFA receptor tyrosine kinase inhibitor (blocks signal transduction through both FLT1 and KDR) significantly reduces vascular density, increases the percentage of primordial follicles and decreases the percentage of developing follicles compared with control ovaries (Fig. 3; McFee et al. 2009). In contrast, treatment with a NRP1 inhibitor (V1; Starzec et al. 2006) only minimally reduces vascular density and does not alter the percent of primordial vs. growing follicles in treated ovaries compared with controls (McFee et al. 2009). However, the percentage of early primary follicles is reduced and the percentage of primary follicles is increased (Fig. 3; McFee et al. 2009). This suggests that VEGFA helps to promote the activation of primordial follicles from the resting state but that this regulation does not appear to require NRP1 binding, although VEGFA binding to NRP1 might help stimulate the development of follicles to the primary state. Furthermore, the localization of VEGFA and its receptors to non-vascular cells, together with the alterations of early follicular development with and without reduced vascularization, indicate that VEGFA regulation of follicle development does not necessarily depend on vascular mechanisms.

Further experiments with the same P3/4 rat ovary culture system demonstrated that treatment with recombinant VEGFA_165 or a VEGFA_XXXB antibody (neutralizes all antiangiogenic VEGFA isoforms) resulted in an increase in vascular density. In addition, treated ovaries had a decreased percentage of primordial follicles and an increased percentage of developing follicles compared with controls (Fig. 3; Artac et al. 2009). Both experiments lend further support for VEGFA’s role in promoting follicle activation and early development. However, neutralization of the antiangiogenic VEGFA_XXXB isoforms produces more pronounced changes than the administration of excess proangiogenic isoforms. This suggests that the antiangiogenic isoforms have a prominent role in regulating follicle progression.

Other studies utilizing postnatal rat ovary cultures have demonstrated a role for estrogen (E_2_) and progesterone (P_4_) in initial follicle recruitment. Approximately half the number of primordial follicles transitioned to primary follicles in PO rat ovary cultures that were treated with E_2_ or P_4_ compared with control ovary cultures (Kezele and Skinner 2003). Circulating levels of E_2_ and P_4_ are high in the developing embryonic and neonatal rat but drop substantially within 2 days after birth (Kezele and Skinner 2003; Montano et al. 1995; Weisz and Ward 1980). In larger mammals, steroid concentrations decrease around the same time that follicles begin to develop (Thau et al. 1976). Taken together, these data suggest that E_2_ and P_4_ can help prevent premature primordial follicle recruitment in embryonic and postnatal development (Kezele and Skinner 2003).

Another study supports this role of E_2_ and also suggests that E_2_ regulates ovarian expression of VEGFA, which promotes follicle recruitment. Intrabursal injection of diethylstilbestrol (DES) in prepubertal (P21) rat pups significantly increases the number of both primary and small secondary follicles in the treated ovary compared with the contralateral ovary. Intrabursal injection with recombinant VEGFA has similar effects on follicle dynamics. In the same study, both systemic and intrabursal administration of E_2_ is shown to result in an increase in ovarian VEGFA protein expression (Danforth et al. 2003).

## Preantral and antral follicle development

The initiation of follicle growth is characterized by two distinct phases. The first is the transition from primordial to primary follicle at which time squamous pregranulosa cells transform into cuboidal granulosa cells. The next phase includes an increase in the number of granulosa cells together with an increase in oocyte size (Braw-Tal and Yossefi 1997). Secondary, or preantral, follicles are classified as an oocyte surrounded by more than two granulosa cell layers (Parrott and Skinner 1999). Complete zonae pellucidae develop in large preantral follicles (Braw-Tal and Yossefi 1997). Early antral follicles have patchy, fluid-containing spaces that lie between granulosa cells and follicles are classified as antral, or tertiary, when the fluid spaces develop into a larger cavity (Smitz and Cortvrindt 2002). In cattle, antral formation first occurs in follicles with at least 250 granulosa cells in the largest histologic cross section (Braw-Tal and Yossefi 1997). Theca cells initially begin to associate with small preantral follicles but a distinct theca layer is only apparent in late preantral or early antral follicles (Braw-Tal and Yossefi 1997). The first theca cells form the theca interna layer which is located closest to the granulosa cells and as development progresses, a theca externa layer forms, which is highly vascularized. It takes a few weeks for an antral cavity to develop after initial follicle recruitment in mice and rats. The same process takes approximately 2 months in humans and several months in large domestic animals (Smitz and Cortvrindt 2002).

Granulosa cells begin to develop follicle-stimulating hormone (FSH) receptors during the preantral stage and theca cells express luteinizing hormone (LH) receptors as soon as they form (Roy et al. 1987; Smitz and Cortvrindt 2002; Sokka et al. 1996). The follicles that are initially recruited and begin development in the prepubertal period never ovulate and are lost via atresia. Cyclic follicle recruitment begins after puberty, because of the increase in circulating FSH levels and ovulation of the first follicle coincides with the first LH surge (Mazaud et al. 2002). During cyclic recruitment, a cohort of antral follicles is able to escape atresia because of the survival action of FSH (Chun et al. 1996; McGee and Hsueh 2000; Scheele and Schoemaker 1996). Loss of gonadotropins via hypophysectomy or follicle culture without hormonal treatments eventually leads to atresia and apoptosis of developing rat follicles (Chun et al. 1996; Nahum et al. 1996). FSH treatment but not LH or human chorionic gonadotrophin (hCG) treatment, can significantly prevent follicular atresia in cultured rat follicles (Chun et al. 1996). Each growing follicle has a threshold requirement for stimulation by FSH and this threshold needs to be surpassed to achieve continued development. The recruited cohort of follicles represents a group of follicles that is at a comparable stage of development and, thus, has similar developmental requirements (Fauser and Van Heusden 1997).

In postnatal rat ovaries, VEGFA, FLT1 and KDR have been localized to granulosa cells, theca cells and the cytoplasm of oocytes of preantral follicles (McFee et al. 2009). In postnatal mice with KDR-LacZ-expressing cells, KDR expression is also seen in granulosa cells, theca cells and the cytoplasm of oocytes within secondary follicles (Bott et al. 2010). Other studies have also demonstrated strong VEGFA immunostaining in secondary and antral follicles from rat ovaries, especially in the theca cells (Celik-Ozenci et al. 2003). In an analysis of human ovaries, VEGFA has been localized to theca interna cells throughout follicle development and to granulosa cells after the primary stage (Yamamoto et al. 1997). Another study has identified immunostaining for VEGFA and FLT1 in the granulosa cells of all developing follicles and in theca cells of medium and large antral follicles (Otani et al. 1999). VEGFA protein is also expressed in bovine fetal ovaries and is predominately localized to blood vessels and secondary follicles. In adult bovine ovaries, immunohistochemistry has localized VEGFA expression to both theca and granulosa cells from preovulatory follicles. KDR expression has been predominately located in granulosa cells but some expression is present in theca cells (Berisha et al. 2000; Greenaway et al. 2004).

Expression of mRNA for *VEGFA_121*, *VEGFA_165*, *VEGFA_189*, *FLT1* and *KDR* has also been demonstrated in fetal bovine ovaries and levels of *VEGFA_121* and *VEGFA_189* increase as development proceeds. Messenger RNA levels for *VEGFA* are consistent across follicle development in theca cells, whereas the expression of *KDR* and *FLT1* is weak in granulosa cells but strong in theca cells (Yang and Fortune 2007). Both granulosa and theca cells in developing follicles from adult bovine and porcine ovaries express mRNA for the *VEGFA_165* and *VEGFA_121* isoforms and the expression of *VEGFA* increases as antral follicles increase in size (Berisha et al. 2000; Mattioli et al. 2001). Cultured bovine granulosa cells predominately express the *VEGFA_120* and *VEGFA_164* isoforms but also weakly express mRNA for *VEGFA_189*. These cultured cells additionally express mRNA for *KDR* (Greenaway et al. 2004). In marmoset monkeys, mRNA for *VEGFA* is expressed in both granulosa and theca cells of secondary and tertiary follicles, whereas *FLT1* and *KDR* are expressed by the endothelial cells within the theca layer in marmoset monkeys. The mRNA levels for *VEGFA* in granulosa cells increase from the secondary follicle stage to the tertiary stage (Wulff et al. 2001, 2002).

VEGFA protein in follicular fluid and granulosa cells has been demonstrated to increase in both bovine and porcine follicles as they increase in size from small to large antral follicles (Berisha et al. 2000; Greenaway et al. 2004; Mattioli et al. 2001). Protein levels of KDR also increase significantly between small and large bovine antral follicles and protein levels in theca cells are similar to those in granulosa cells from large antral follicles (Greenaway et al. 2004). Medium antral follicles from gilts have distinct differences in VEGFA protein expression. Those follicles with high follicular fluid concentrations of VEGFA also have high follicular fluid levels of E_2_ and wider vascular networks within the follicular wall than medium follicles with low VEGFA levels (Mattioli et al. 2001).

Based upon mRNA and protein expression alone, VEGFA appears to be involved in the growth of follicles from the preantral and early antral stages to later antral stages. Numerous other studies have added support for the role of VEGFA in follicle development. Culturing pieces of bovine fetal ovarian cortex in the presence of VEGFA has no effect on the number of primordial or primary follicles but does increase the number of secondary follicles (Yang and Fortune 2007). Injection of *VEGFA* gene fragments into the ovaries of miniature gilts results in increased numbers of large antral follicles, increased mRNA expression of the *VEGFA_165* and *VEGFA_121* isoforms in granulosa cells and increased VEGFA protein levels in follicular fluid. In addition, the capillary density within the theca interna is increased in follicles from VEGFA-injected gilts compared with control animals (Shimizu 2006; Shimizu et al. 2003a). Intramuscular injection of prepubertal gilts with eCG induces an increase in VEGFA protein levels in follicular fluid and *VEGFA* mRNA levels in granulosa cells of follicles larger than 4 mm in diameter (Barboni et al. 2000). VEGFA treatment also results in an increased number of preovulatory follicles, a decreased number of atretic follicles and an increased number of ovulated oocytes (Iijima et al. 2005).

VEGFA regulation of preantral and antral follicle growth appears to be mediated through FLT1 and KDR signaling. Inhibition of VEGFA with intrabursal injection of a soluble FLT1/Fc chimera Trap does not alter the number of preantral or early antral follicles in prepubertal rats treated with eCG. However, the number of atretic follicles increases compared with control rats, together with increased BAX and decreased BCL2 protein levels in follicular cells (Abramovich et al. 2006). Neutralization of VEGFA by the administration of a VEGF Trap (truncated versions of FLT1 or FLT1 and KDR fused to IgG) significantly reduces granulosa cell proliferation, theca proliferation and thecal vascularization in secondary and tertiary follicles in marmoset monkeys (Wulff et al. 2001, 2002).

## Concluding remarks

**Fig. 4:**
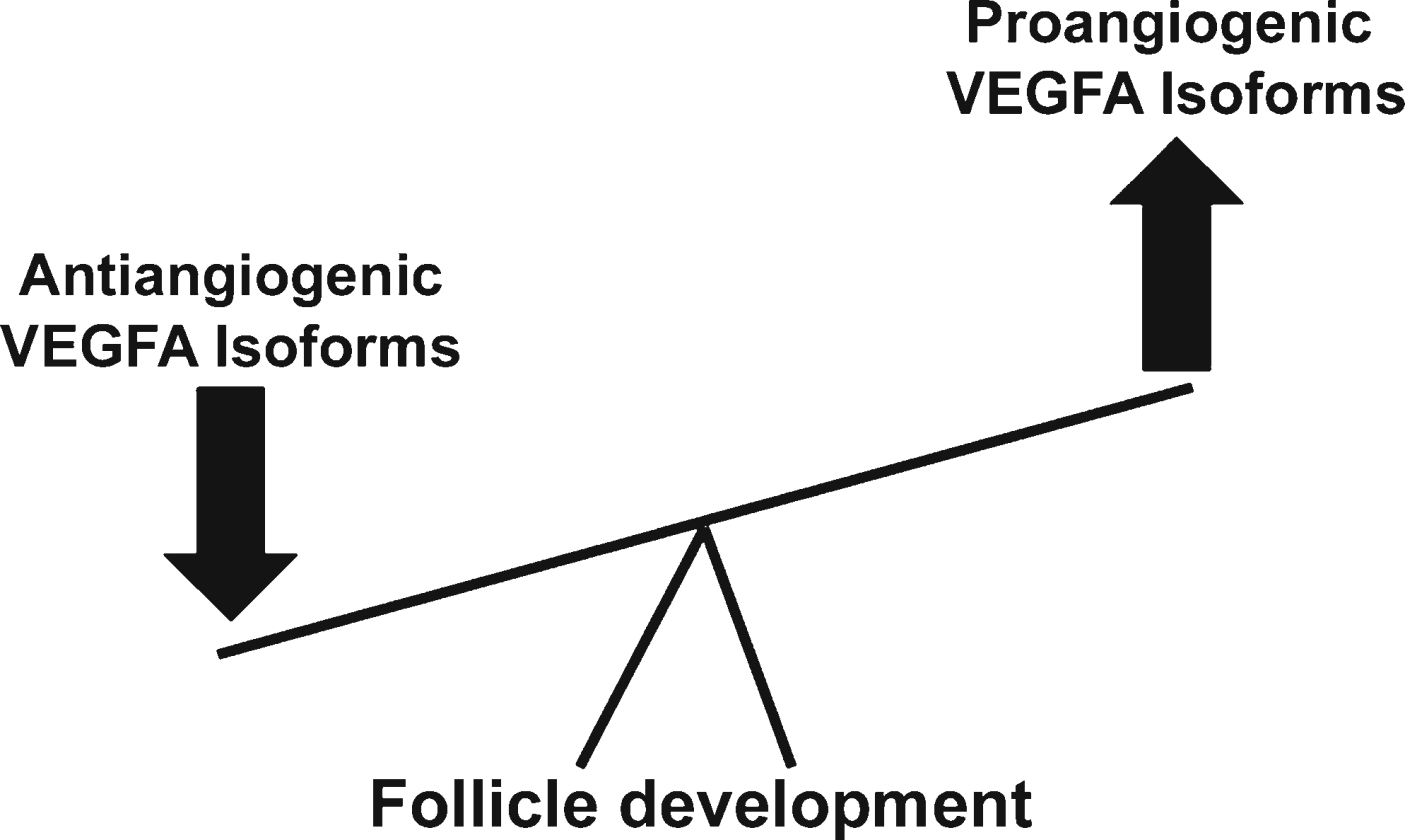
Representation of the proposed role for VEGFA isoforms in follicle development. Proangiogenic isoforms appear to promote initial recruitment and development of ovarian follicles, whereas antiangiogenic isoforms appear to suppress these processes

A strong body of evidence supports a role for VEGFA in initial follicle recruitment and development. Even though VEGFA is considered a prominent proangiogenic factor, VEGFA has been localized to nonvascular follicles and cells and might influence follicular development via nonvascular mechanisms. In addition, VEGFA isoforms have now been identified that have antiangiogenic properties (Harper and Bates 2008). Specific agents have been developed that differentiate between the antiangiogenic and proangiogenic VEGFA isoforms; however, many of the studies evaluating VEGFA in regard to ovarian and follicular development up until this point have not differentiated proangiogenic VEGFA from antiangiogenic VEGFA. One must take this into consideration when interpreting the findings from these studies. In vitro experiments from our laboratory indicate that proangiogenic VEGFA promotes, whereas antiangiogenic VEGFA suppresses follicle recruitment and early follicular development (Fig. 4). Further studies are warranted to elucidate the way in which the different VEGFA isoforms regulate follicular formation and progression. In vivo experiments utilizing cell-specific mutant models that lack or overexpress proangiogenic and antiangiogenic isoforms will further define the role of VEGFA in these processes and aid our understanding of the balance of proangiogenic versus antiangiogenic VEGFA isoforms in follicular development.
